# Experimental study on the failure process and modes of loess spoil slope induced by rainfall and engineering disturbance

**DOI:** 10.1371/journal.pone.0305871

**Published:** 2024-07-18

**Authors:** Wei Xiao, Weiping Tian

**Affiliations:** 1 School of Civil Engineering, Chang’an University, Xian, China; 2 Key Laboratory for Special Area Highway Engineering of Ministry of Education, Chang’an University, Xian, China; China University of Mining and Technology, CHINA

## Abstract

In this paper, indoor model tests were conducted using image analysis, pore pressure, and displacement measurement methods to investigate the failure evolution process and modes of loess spoil slopes with various components under the influence of rainfall and artificial excavation. The results of the experiments reveal that, under the action of rainfall, there are two types of cracks-to-failure modes for pure loess spoil slopes. One involves the formation of a large gully through the dominant channel, while the other is characterized by step-by-step retreating soil damage between cracks. The failure exhibits three distinct stages, and after failure, the slope angle is relatively large (>45°). The process of rainfall-induced destruction affecting loess spoil containing 25% coarse-grained content similarly unfolds in three stages, ultimately resulting in the formation of a regional landslide. This landslide typically encompasses a broader damage range compared to pure loess spoil, albeit with a shallower depth of damage. After the landslide stops and stabilizes, a tiny slope (45°) is created (<45°). The excavation at the toe of the slope induces loess spoil damage in a progressive multi-stage receding manner. This study provides a reference and basis for disaster prevention and warning of spoiled ground in loess areas.

## Introduction

Spoil sites, as a byproduct of road construction, are extensive and widespread in China’s mountainous areas, railways, and urban development. In particular, the loess spoil site is a substantial accumulation of loosely packed gravelly silt, primarily composed of loess and formed during engineering construction in loess regions. Its uncompressed and loose structure renders it highly prone to deformation and instability when subjected to external environmental factors [1].

Early constructed spoil sites were frequently in disrepair and functionally impaired, leading to incidents such as landslides during extreme or sustained rainfall. These occurrences posed a significant threat to major infrastructure and the safety of residents and property in mountainous towns and cities [2]. The 2015 Shenzhen spoil site landslide resulted in 77 fatalities and the destruction of 33 buildings [3]. Similarly, the collapse of a spoil site in Shuangjing Village, Yufengshan Township, Yubei District, Chongqing City, in December 2023 caused three fatalities. Consequently, the prevention and control of spoil site disasters will continue to be a critical focus for engineering safety and regional disaster reduction efforts in China in the foreseeable future.

Scholars have mainly directed their studies toward the selection of spoil sites [4], stability analysis, evaluation, and monitoring. In recent years, research has also been conducted on the characteristics of dumped soil [1, 5]. From a macroscopic perspective, two main factors trigger spoil site damage. The initial factor is water, which includes rainfall [6], irrigation [7–9], groundwater, and even stagnant water that was not completely discharged before the spoil site was put into operation [3]. The infiltration of dynamic water significantly contributes to the damage of soil spoil slopes [10, 11]. The second factor affecting the stability of the spoil slope is engineering disturbance. This involves excavating at the base of the slope or loading piles at the top. Engineering disturbances modify the original stress state of the slope, leading to an expansion and loosening of the current structural surface [10].

Spoil sites constructed in loess areas contain substantial amounts of loosely piled, uncompacted loess and may also include stony dumps resulting from excavation to bedrock. The gravelly silt exhibits lower structural properties and strengths, such as shear resistance, compared to the original loess that has undergone long-term exposure to self-weight and natural forces. The distinctive structural properties of loess spoil stem from its non-uniform and uncertain composition. This complexity complicates the slope failure behavior of these sites. Furthermore, the dynamic mechanism and movement characteristics of landslide formation in loess spoil sites are not yet fully understood.

Scholars have extensively researched the types and characteristics of loess [12–16], the factors influencing and genesis mechanisms of landslides [16], the dynamic characteristics of landslides [10, 17, 18], the monitoring, forecasting, and prevention of landslides [19–22], and landslide susceptibility prediction [23]. Numerous landslides in loess areas have demonstrated that under varying rainfall and geological conditions [24, 25], loess slopes may remain stable, experience shallow localized landslides, or undergo high-speed remote landslides [26–28]. Research has shown that materials with a coarse-grained structure, large pores, and weak cementation characteristics may exhibit shear shrinkage effects and collapse instability when subjected to constant load and moisture addition coupling [27, 29].

Unsaturated soils can exhibit significant hydraulic-force coupling effects under loading [30]. Scholars have conducted numerous experimental and theoretical studies on the hydraulic stress characteristics of unsaturated soils. For instance, Cai et al. conducted unilinear triaxial humidification tests on unsaturated sandy loess and investigated the effects of hydraulic-force paths and stress ratios on deformation characteristics during humidification [31]. Zhang et al. performed triaxial shear tests on unsaturated soils using a penetrometer. They analyzed the effect of hydraulic paths on the moisture-enhanced shear characteristics of in situ loess and the necessary conditions for the occurrence of wet shear damage [32]. While these studies primarily focused on obtaining wetting and deformation characteristics of soils, such as clays and chalks, with finer particles and smaller pore sizes through shear tests, there have been relatively few studies conducted on the deformation-damage patterns of broadly graded gravelly soils, gravelly silt, and other soils considering different particle gradation factors.

Scholars also conduct studies on the impact of rainfall on loess humidification. Yang et al. revealed the soil-water characteristic curves and the hydraulic coupling process of deformation damage of landslide accretion under rainfall-excited conditions through rainfall tests on unsaturated accretionary soils [33]. Wei et al. proposed that the decreasing matric suction in response to rainfall infiltration at shallow depth is the main trigger for those landslides on planar and concave slopes with a relatively thick soil mantle [34]. According to the model test of Tang et al., the main cause of landslide instability in the slip-resistant section of the loose accumulation is the rapid decrease in effective stress or even local liquefaction of the soil [35]. They reached this conclusion after changing the sliding surface inclination and infiltration form. It is important to note that subjective evaluations have been excluded, and technical term abbreviations have been explained when first used. The studies above primarily concentrate on establishing a geological-hydrological-mechanical mechanism model of rainfall-induced slopes. However, there are fewer studies on the changes in the stress state of existing structural surfaces caused by engineering disturbances, the damage modes of the slopes, and the damage modes of the slopes caused by their coupling with rainfall. It is important to note that the improved text must not introduce any new aspects beyond those mentioned in the original text.

Therefore, this paper aims to investigate the effects of rainfall and manual excavation on soil spoil slopes with varying particle grades. The study establishes multiple groups of spoil content components for indoor model tests, using rainfall and slope toe excavation as the two primary factors causing damage. Displacement-time variation curves of the loess spoil slopes during rainfall, post-rainfall, and post-excavation, along with pore pressure-rainfall-time curves, were obtained to identify the behavioral characteristics of loess spoil during rainfall and after excavation. These findings could provide a foundation for disaster prevention and early warning systems for spoil sites in loess areas.

## Indoor model testing of soil spoil slopes

### Preliminary preparations

Commissioned by China Railway Construction Shaanxi Expressway Co., field investigations, field particle sieving tests, natural slope angle tests, and sampling of a large number of spoil grounds along railway and highway routes in the loess region have been conducted. Data on the topography, geomorphology, hydrology, rainfall, and other relevant information in the area where the spoil ground is located have been collected. Additionally, slope angle, slope type, slope height, and the utilization of spoil retaining dams and drainage ditches have also been recorded.

### Model testing facilities

**Fig 1:**
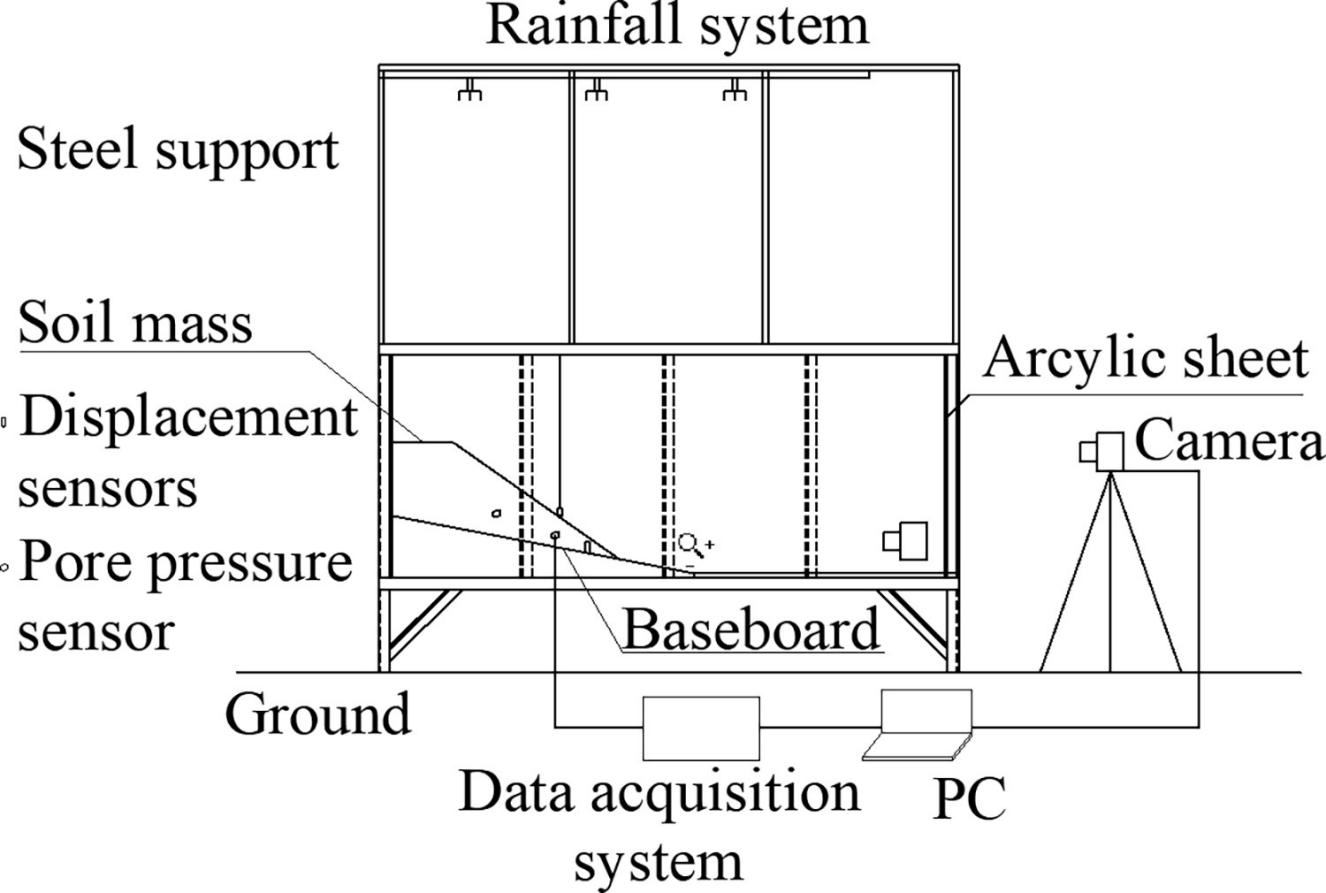
Test system diagram.

**Fig 2:**
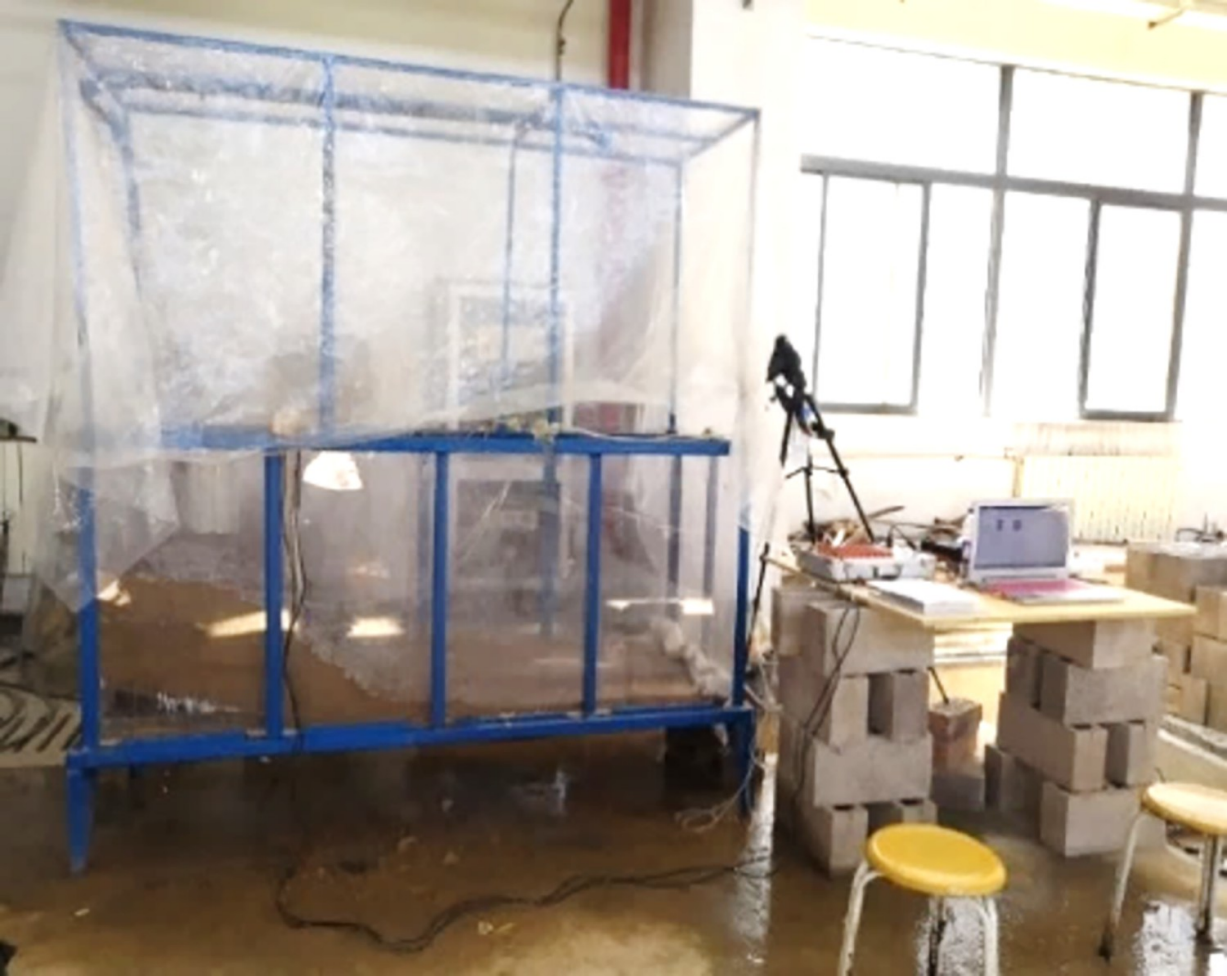
Model test site photos.

**Fig 3:**
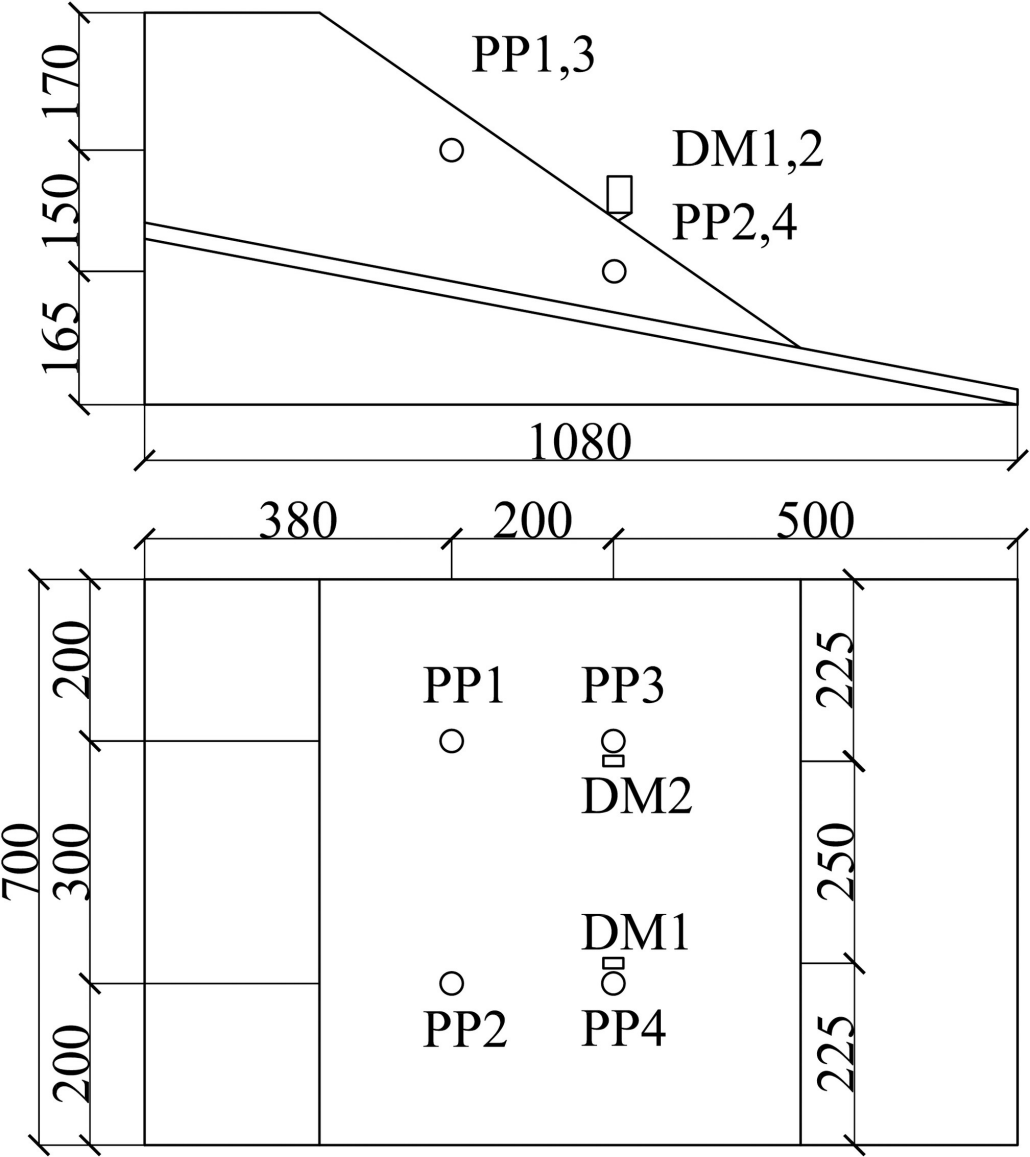
Arrangement of sensors.

In the experiment, an artificial rainfall testing box system consists of a model testing box, a controlled artificial rainfall system, and a visual dynamic measuring system. Fig 1 depicts the system diagram, while Fig 2 shows an on-site photograph of the model box. Fig 3 illustrates the configuration of pore pressure sensors (PP1 to PP4) and displacement meters (DM1/DM2).

1. The model box has dimensions of 0.7 m x 2.0 m x 1.0 m and is constructed with angle steel and tempered glass plates. The top side of the box is open, the bottom side includes a permeable layer and a water outlet, and the sides are transparent.
2. The adjustable artificial rainfall system comprises a flow meter, an inlet system, and five low-pressure atomizing nozzles arranged in a row. The atomizing nozzle has a diameter of 1.0 mm, and the sprayed water droplets are homogeneous, appearing in the form of a mist. The flow meter in the water inlet system adjusts the intensity of the rainfall during the test.
3. The visual dynamic measurement system includes a lighting system, digital image recording equipment, and a remote data acquisition system.
4. The data measurement system consists of four pore pressure sensors (PP1~PP4), two displacement sensors (DM1/DM2), and a data acquisition system. The data acquisition system comprises four parts: data collection, control, transmission, and recording.

### Soil samples

According to the survey results, the spoil is categorized into three groups based on the content of coarse-grained soil with soil diameters greater than 2 mm: stone spoil (coarse-grained content ≥ 75%), soil spoil (coarse-grained content ≤ 25%), and mixed soil and rock spoil (25% < coarse-grained content < 75%).

**Fig 4:**
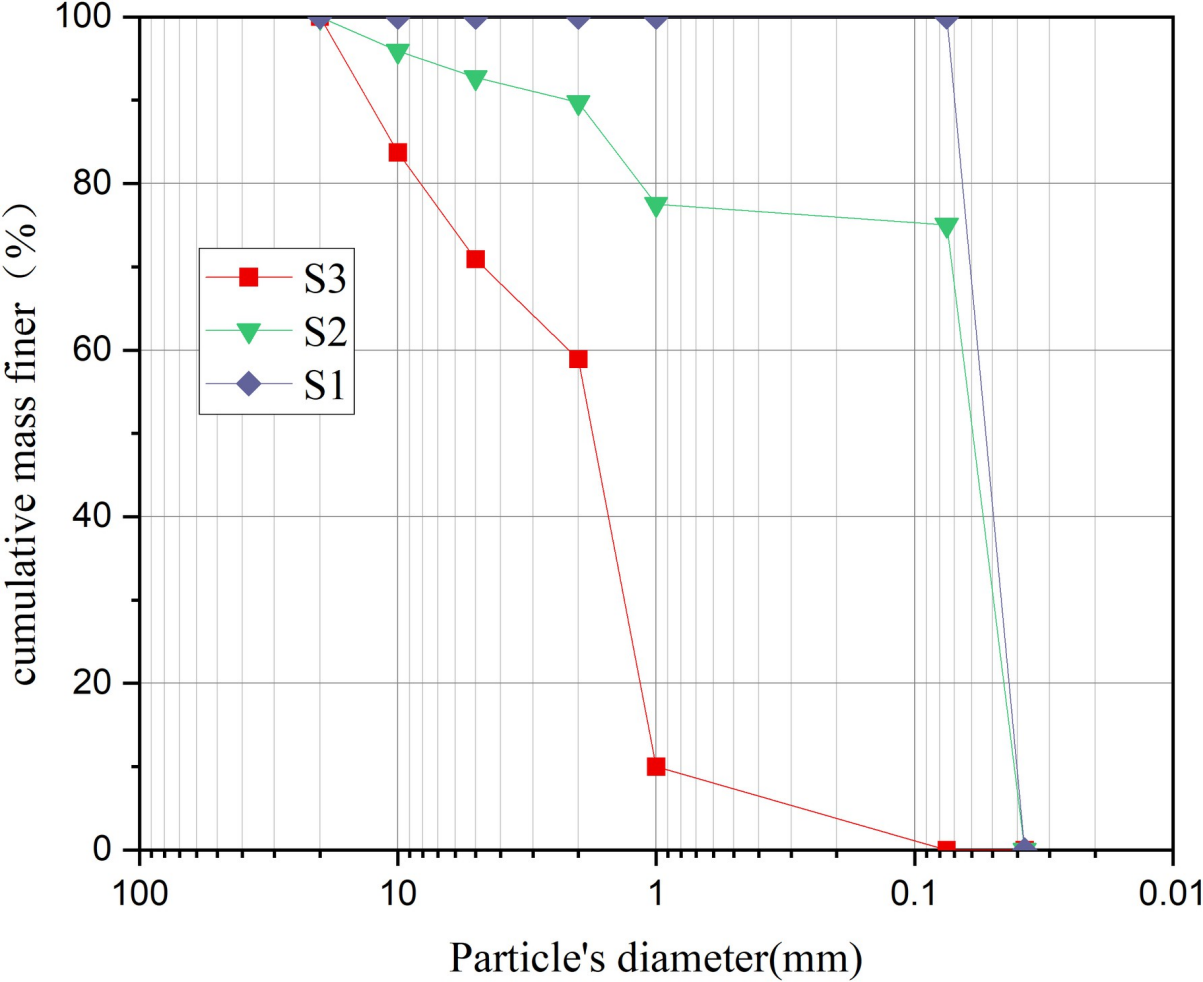
Particle grading curve of soil samples.

In this test, Xi’an loess was utilized as soil sample 1 (S1), representing a pure loess spoil group with a coarse-grained soil content of 0%, marking the lower limit of the soil spoil. A soil sample from a typical stony dump site was chosen, where particles above 60 mm were removed, followed by a 2:1 reduction. This process resulted in stony dump soil sample S3 (with 100% stone content), serving as the control group for the test. Two types of soil samples were prepared with a coarse-grained soil content of 25%, yielding spoil soil sample 2 (S2), representing the upper limit of soil spoil. The grading curves of the test soil samples are presented in Fig 4.

With a coarse-grained soil content of 0%, marking the lower limit of soil spoil, Xi’an loess was employed as soil sample 1 (S1) in this test. A soil sample from a typical stony dump site was selected, and after removing particles larger than 60 mm and completing a 2:1 reduction, stony spoil soil sample 3 (S3) containing 100% coarse-grained content was acquired and served as the control group for the test. To create spoil soil sample 2 (S2), representing the upper limit of soil spoil, the above two types of soil samples were configured to have a 25% coarse-grained soil content. Fig 4 illustrates the grading curves for the test soil samples.

S1’s strength is determined through a direct shear test, while S3’s strength is determined using a large direct shear test. Table 1 displays the characteristics of the soil samples.

**Table 1:** Characteristics of soil samples for indoor test.

	Coarse grained soil(>2mm) content (%)	Maximum dry density(g/cm^3^)	Minimum dry density(g/cm^3^)	Mass moisture content(%)	C_u_	C_c_	Effective internal friction angle(°)	Effective cohesion(kPa)
**S1**	0	1.32	1.231	1.54	1.231	0.988	15	27
**S2**	25	1.54	1.454	1.06	1.385	0.962		
**S3**	100	1.938	1.848	0.254	2.02	1.092	44.9	0

### Test conditions and parameters

Continuous concentrated rainfall, with an intensity of 272 mm/h, was employed during the experiment. The rainfall ceased after the destruction of the spoil slope, and the slope stabilized for some time. The angle between the slope and the horizontal plane is 40°.

### Test protocols

Two stages of testing were conducted on all three soil samples. The first stage is the pure rainfall stage, and the second stage involves excavating the toe of the slope after the initial stability and maintaining rainfall, inducing secondary damage until stabilization is achieved again. The specific test parameters and protocols are presented in Table 2, with the test number represented as Tn-s, where T denotes the test, n denotes the test number, and s denotes the testing stage.

**Table 2:** Indoor test parameters and protocols.

Test NO.	Soil sample type	Coarse-grained soil (>2mm) content (%)	Particle content above 0.5mm(%)	dry density(g/cm^3^)	Mass moisture content(%)	Factors of inducing damage
**T1-1**	S1	0.0	0	1.284	1.540	rainfall
**T1-2**	S1	0.0	0	1.284	1.540	Toe excavation
**T2-1**	S2	10.3	25	1.463	1.060	rainfall
**T2-2**	S2	10.3	25	1.463	1.060	Toe excavation
**T3-1**	S3	41.1	100	1.897	0.254	rainfall
**T3-2**	S3	41.1	100	1.897	0.254	Toe excavation

## Evolution process of loess spoil slope failure test

### Final form of loess spoil slope failure

**Fig 5:**
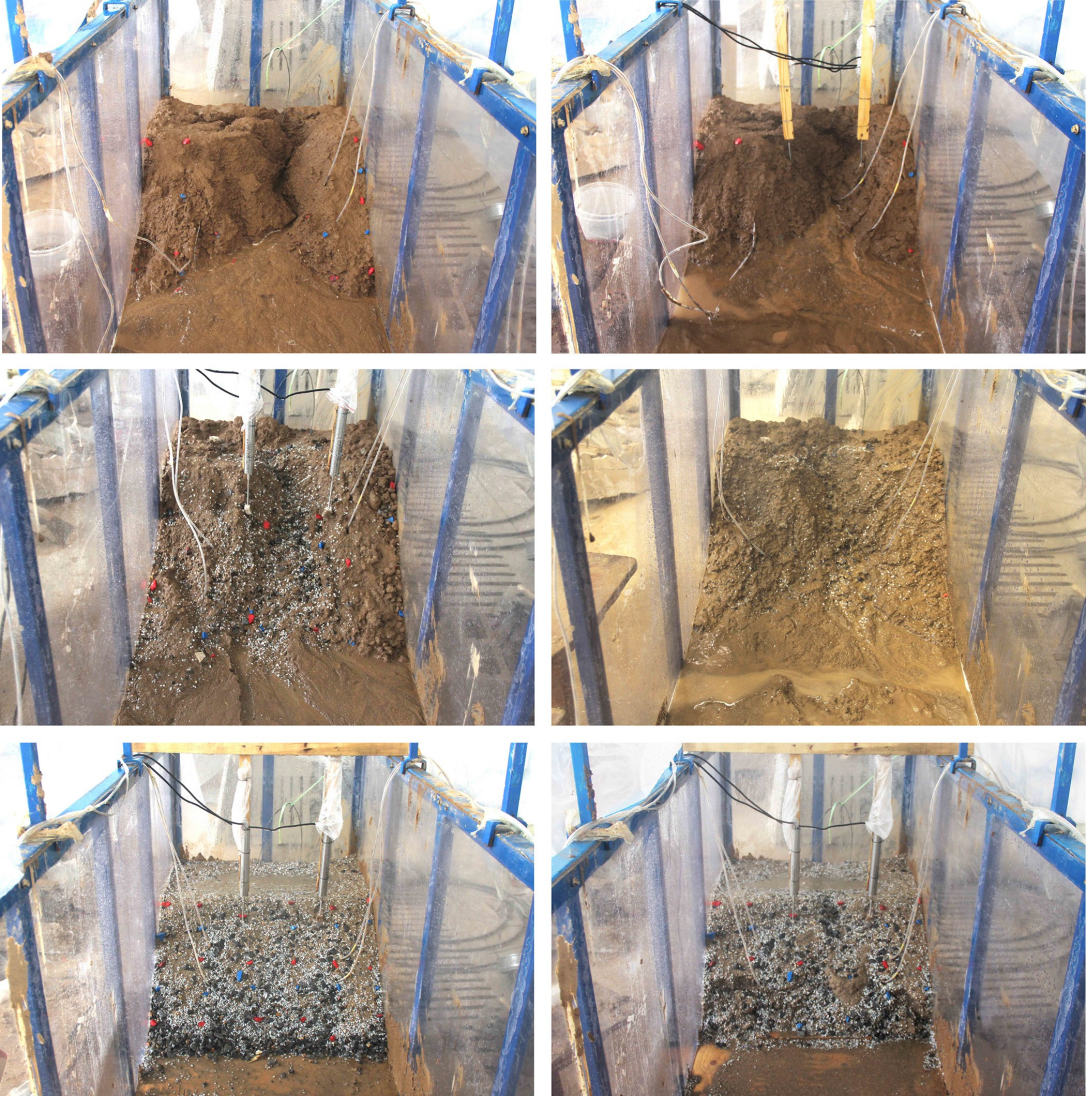
Slope ultimate failure shape for each test. (a) T1-1, (b) T2-1, (c) T3-1, (d) T1-2, (e) T2-2, (f) T3-2.

Using a digital camera, the rainwater penetration and slope deformation processes inside the model box were recorded during the experiment. Fig 5 illustrates the final failure shapes of three soil samples tested in six groups. Fig 5(A)–5(C) depict the ultimate failure patterns of slopes induced by rainfall for three soil samples, ranging from S1 to S3. Fig 5(D)–5(F) show the final failure patterns of slopes generated by excavation at the slope’s foot and then maintaining rainfall for three soil samples, also ranging from S1 to S3.

### The failure process of soil samples under different induced conditions

#### Evolution mode of spoil slope failure induced by rainfall

*(1) S1 spoil (pure loess sample with a coarse-grained content of 0%)*. Test 1–1 (T1-1) illustrates rainfall-induced loess spoil slope failure, specifically a nibbling retreat failure. Under the influence of rainfall, two distinct types of cracks-to-failure evolution modes with three stages of features are observed in various slope regions, including weak areas and other locations.

**Fig 6:**
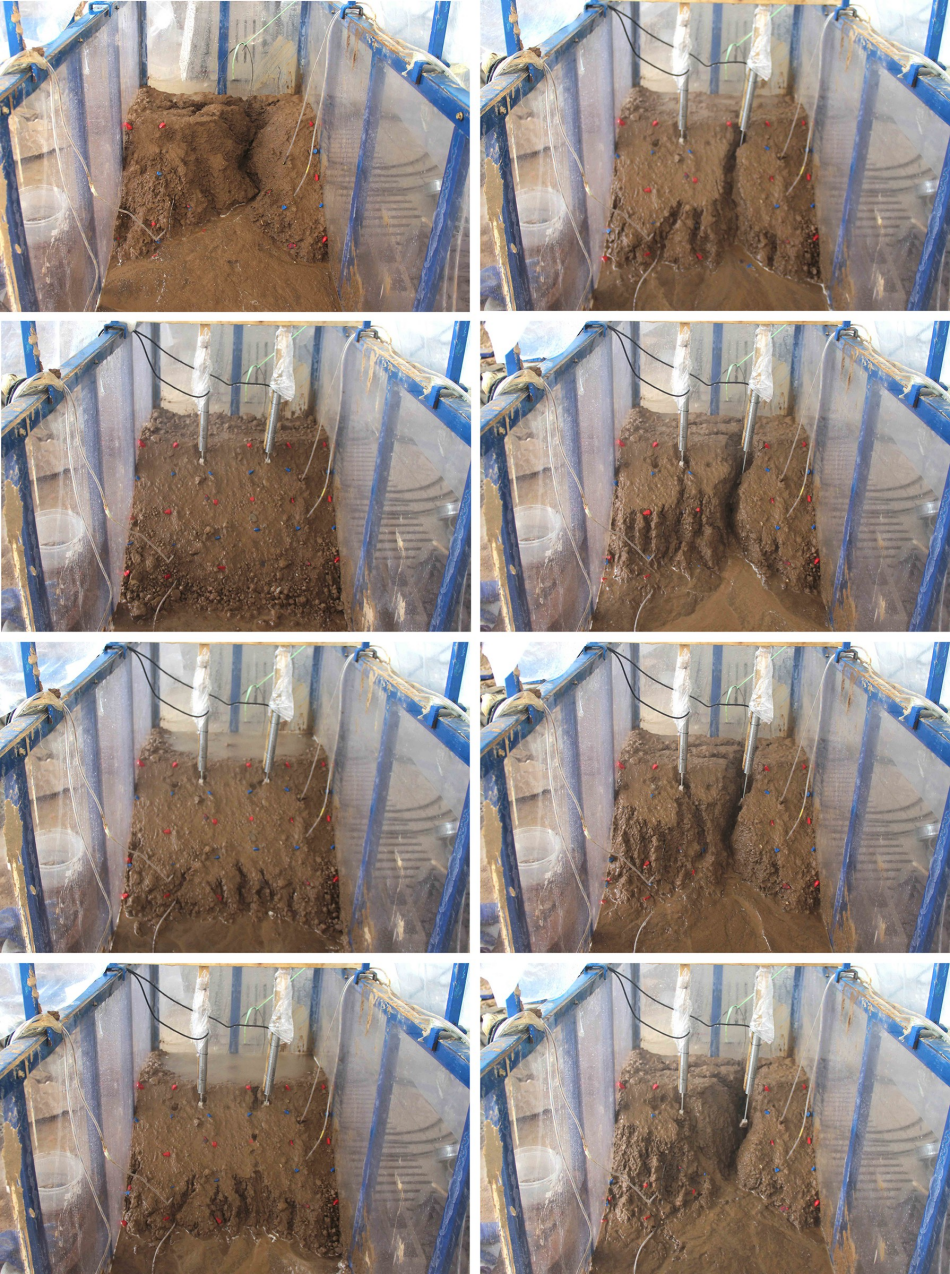
Test T1-1 progress. (a) 1254s Initial state, (b) 1794s Cracks appear at the toe of the slope, (c) 2034s Crack propagation, (d) 2334s Cracks run through to the top of the slope, (e) 4434s Gradual retreat failure of soil between slope toe, (f) 5694s Collapse at the toe of the slope, forming another continuous gully, (g) 7614s Collapse of soil between gullies, (h) 12354s Gully edge extension.

In the first stage, a portion of the rainwater flows along the slope towards the lower part, while the remaining rainwater, not drained promptly, gradually infiltrates downward from the loess surface to the deeper layers. When the sum of infiltration and drainage is insufficient to match the rainfall, water accumulates on the top surface of the waste slag. The soil layer at the foot of the slope is the thinnest and saturates first, leading to the earliest appearance of cracks (Fig 6B).

In the second stage, the soil in different areas on the slope evolves in varying directions due to the high porosity resulting from the loose accumulation of spoils. As pore water pressure rises, cracks emerge at the top of the soil mass when water accumulates in vulnerable places. Rainwater infiltrates through these cracks, and eventually, they converge and penetrate at a specific time, creating a dominant channel that runs directly from the top to the bottom of the slope. This channel is continually eroded and widened by the rainwater, forming the deepest and largest gullies on the slope, representing the first type of crack-to-failure evolution.

Meanwhile, at the toe of the spoil slope, several vertical cracks had already developed in other areas. The constant rains caused the fractures to enlarge and lengthen, resulting in increased soil particle loss and decreased distance between the cracks. The soil’s horizontal stress decreases between the cracks, leading to a rise in deviatoric stress (σ1 - σ3). The soil undergoes shear collapse, and minor local landslides develop, forming the second type of crack-to-failure evolution.

In the final stage, the slope’s toe collapses, and a sizable gully forms deep in the ground. The first type of crack-to-failure development led to the formation of the enormous gully, while the second type of crack-to-failure evolution resulted in slope toe collapse.

**Fig 7:**
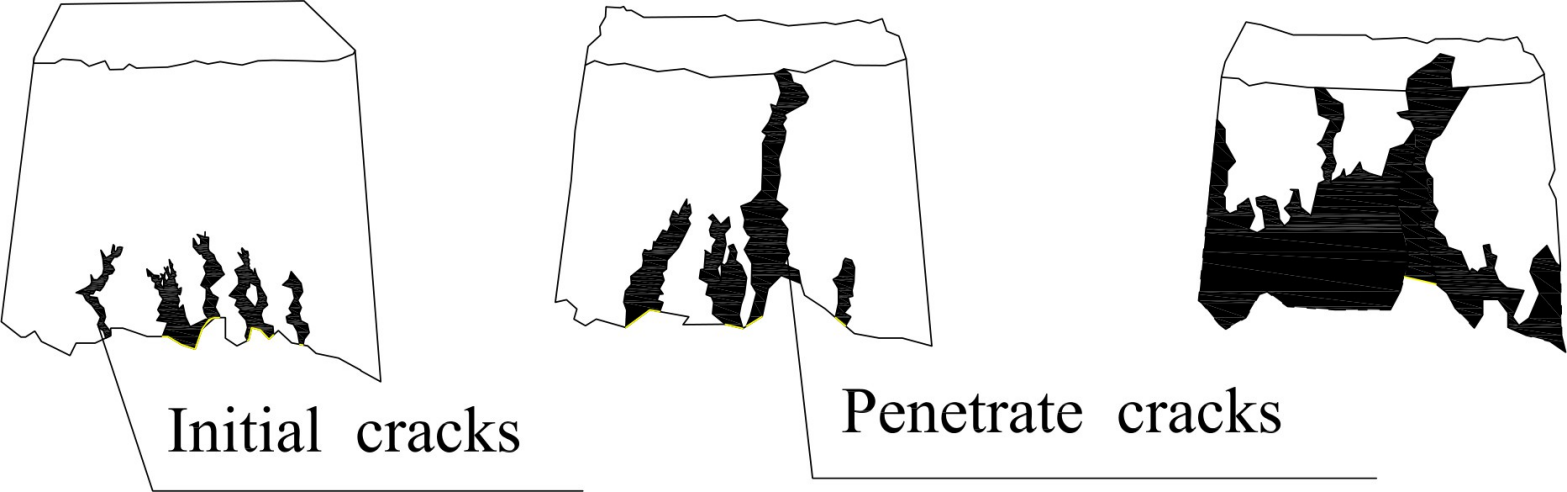
Schematic diagram of the development process of pure loess spoil slope failure induced by rainfall.

The complete failure process generally unfolds as follows: ① the toe of the slope develops small vertical cracks; ② the dominant channel evolves from a dominant vertical crack into gullies. Simultaneously, several cracks gradually extend upward from the toe of the slope, causing the soil between the foot of the slope to retreat and fail; ③ the gullies expand through the soil mass, and the toe of the slope collapses. Fig 6 illustrates the failure process, while Fig 7 provides a schematic representation of the three stages of the failure process.

The saturation of the surface soil at the toe of the slope is the initial indication that the loess spoil slope is prone to failure. Damage may occur in this region when the loess at the slope’s surface becomes wet, gradually reaching saturation, and is subsequently washed away due to continuous rainfall. Following the landslide’s cessation and stabilization, a relatively steep slope (>45°) forms, exhibiting specific characteristics of loess.

**Fig 8:**
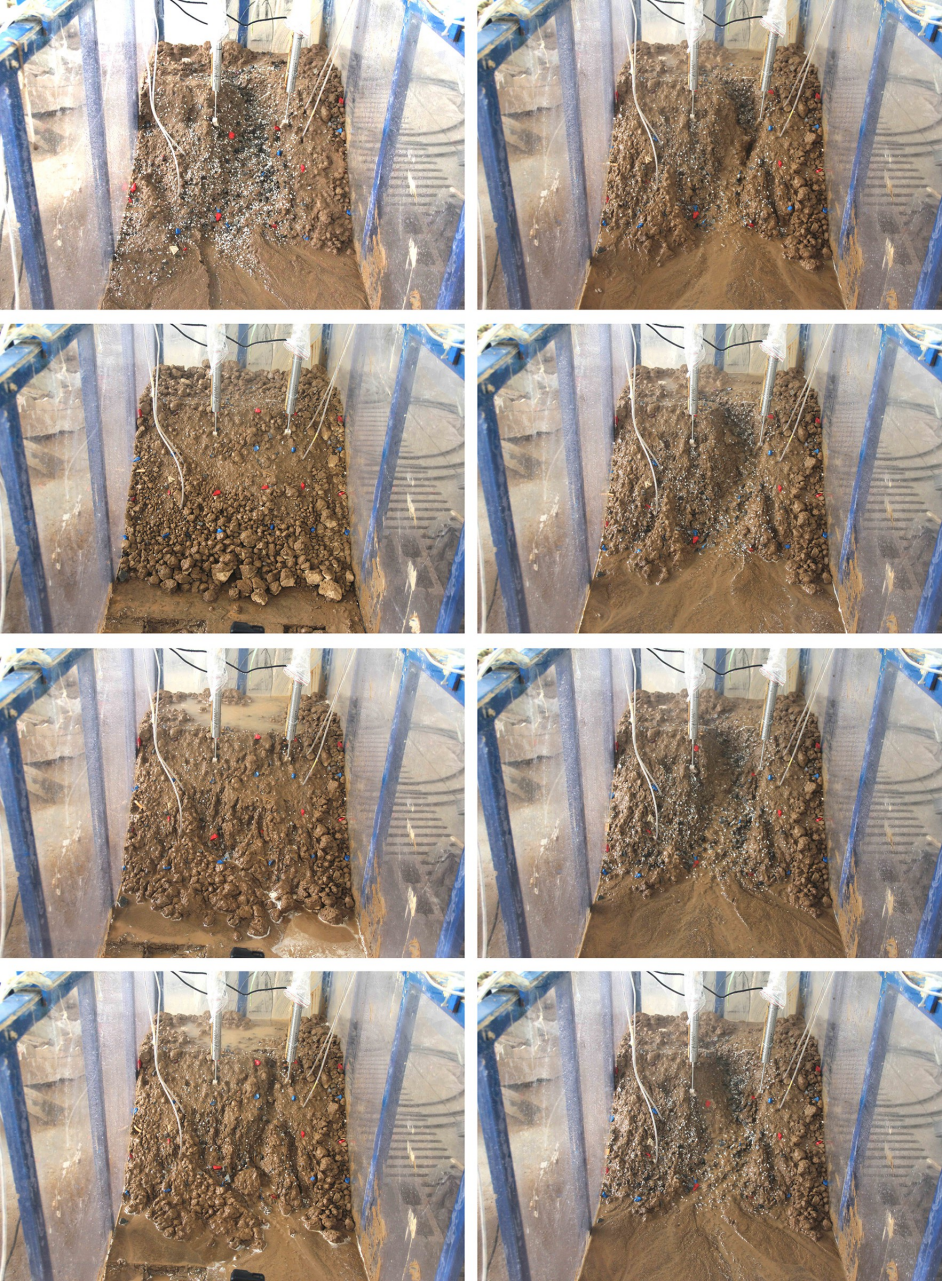
The process diagrams of T2-1. (a) 1740s Initial state, (b) 2340s Cracks appear at the toe of the slope, (c) 2520s Cracks run through to the top of the slope, (d) 4200s Gully is forming, (e) 4560s Gully Continuous widening, (f) 6360s Gully Continuous widening, (g) 7260s Forming a regional landslide, (h) 18060s Small particles of loess are carried away by water.

*(2) S2 spoil (coarse-grained content*: *25%)*. The procession of slope failure is separated into three stages as well:

Initially, similar to the loess spoil slope, water will collect on the top surface of the spoil mass, and multiple cracks will emerge near the toe of the slope (Fig 8B). The cracks widen with the continuous rainfall.

Subsequently, water gushes out from low-lying areas on the top of the slope, forming a top crack that connects to the bottom crack, creating a large gully. The gully then deepens and widens on both sides due to the influence of rainwater. Larger particles remain on the surface after the water flow carries away smaller particles from cracks. These cracks combine to form several small gullies.

In the final stage, the main gully begins to erode towards both banks, eventually forming a broad, gradual slope. Shallow valleys in other areas of the slope extend to the top, resulting in a regional landslide.

Fig 8 illustrates the T2-1 failure mode for S2. In contrast to T1-1 for S1, where there is inter-crack regressive damage, large particles are retained in the cracks, and fine loess particles are carried away by running water. This process ultimately results in the creation of a regional landslide with a greater extent of final damage than pure loess spoil, yet with a lesser depth of damage. After the landslide ceases and stabilizes, a gentle slope of approximately 45° is formed.

*(3) S3 spoil (Coarse-grained content*:*100%)*. Stone spoil is the term used to describe this sample. Due to the presence of numerous pores between the particles, it exhibits excellent drainage characteristics. The failure mechanism of the stone spoil slope under rainfall involves localized shallow erosion: the slope typically remains stable, with minimal erosion and loss of particles smaller than 2 mm from the slope surface. Apart from some shallow erosion, there are no gullies or fractures. Fig 5(C) illustrates the final shape of the slope failure.

#### Evolution mode of spoil slope failure induced by slope toe excavation with rainfall

**Fig 9:**
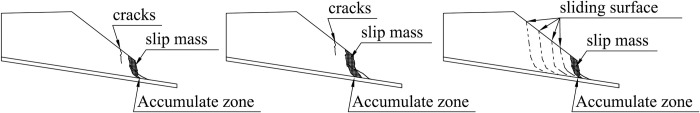
The process schematic diagram of test T1-2.

*(1) S1*. A free surface emerged after the excavation of the slope’s toe. Shear stress increased, leading to the formation of a stress concentration zone, causing the soil to enter a yielding state and resulting in the development of transverse tensile cracks (as depicted in Fig 9). Continuous rainfall weakens the bonding effect between loose spoil particles. Rainwater seeps into the cracks, forming a saturated soft sliding zone at the bottom, and the horizontal cracks rapidly expand. The sliding force of the soil blocks beneath the cracks surpasses the anti-sliding force due to gravity. These blocks quickly detach and accumulate at the slope’s base before being washed away by the water flow.

The slope failure mode involves a progressive multi-stage retreat under the condition of excavation at the slope’s toe, and the failure process unfolds as follows: ① Initial appearance of transverse tensile cracks; ② Subsequent development of cracks towards the deeper part of the soil mass; ③ Transfixion of cracks, leading to the upper part of the soil in the surrounding area peeling off and accumulating at the slope’s toe. Fig 9 illustrates schematic diagrams of the T1-2 test failure process.

**Fig 10:**
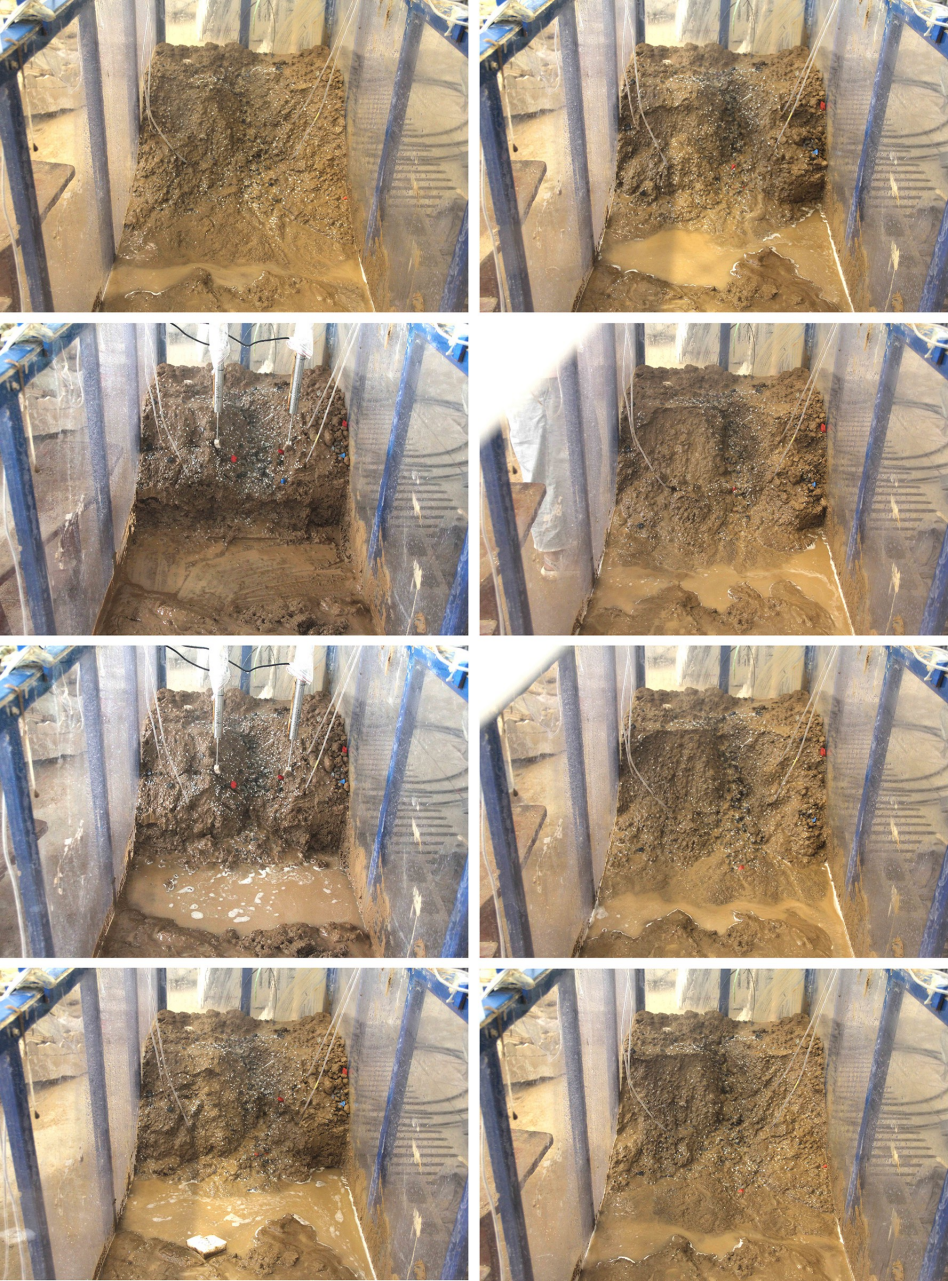
The process diagrams of test T2-2. (a) At 10th sec. Initial state after excavation, slight tension cracks appear, (b) At 70th sec. Local collapse and crack expansion, (c)At 190th sec. The second step collapses and new tension cracks appear, (d) At 370th sec. The collapse range extends backward, (e) At 490th sec. The collapse extends to the top of the slope, (f) At 790th sec. Collapse range lateral expansion, (g) At 1330th sec. Some parts form a gentle slope, (h) At 2110th sec. The original gully area expansion.

*(2) S2*. The failure mode in T2-2, during excavation at the toe of the slope, is characterized by multi-stage regression. In contrast to S1, the tensile cracks are more pronounced at each stage of retreat. Fig 10 illustrates the T2-2 failure process.

**Fig 11:**
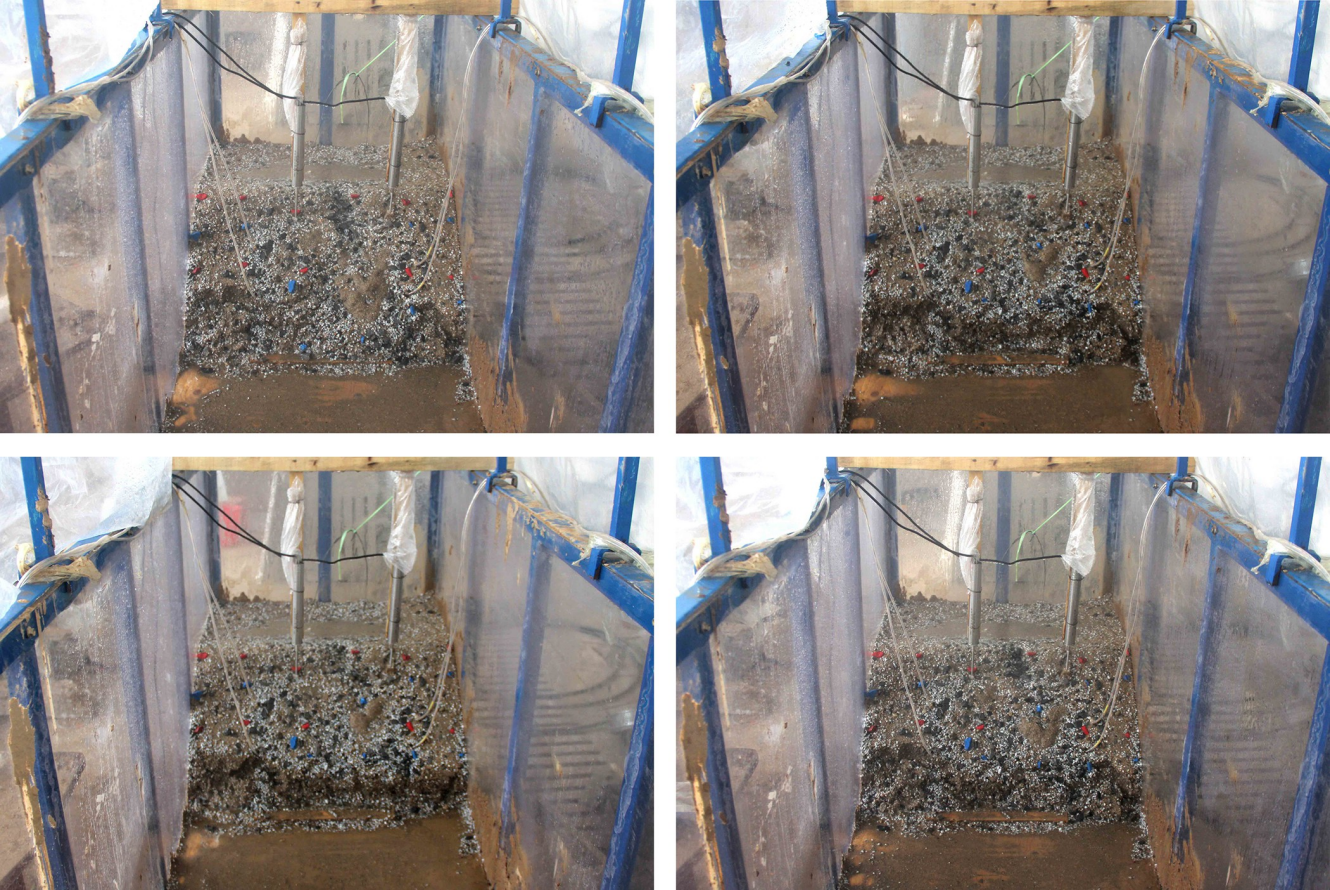
The process diagrams of test T3-2. (a) At 1860th sec. Initial state after excavation slope toe, (b) At 3780th sec. Slight local collapse at the toe of the slope, (c) At 8940th sec. Forming linking cracks, (d) At 12000th sec. Forming a shallow landslide of fan-shaped.

*(3) S3*. Fig 11 illustrates the T3-2 failure stage in the scenario of excavation at the toe of the slope with rainfall. Shallow local sliding is the failure mode.

## Results and analysis of displacement and pore pressure tests

Two displacement meters and four pore pressure sensors were installed inside the soil; their placements are depicted in Fig 3. The displacement sensors monitor the displacement of the points on the slope surface, while the pore pressure sensor measures the pore pressure at a location 12 cm below the slope surface.

### The impact of rainfall on slope surface displacement changes

**Fig 12:**
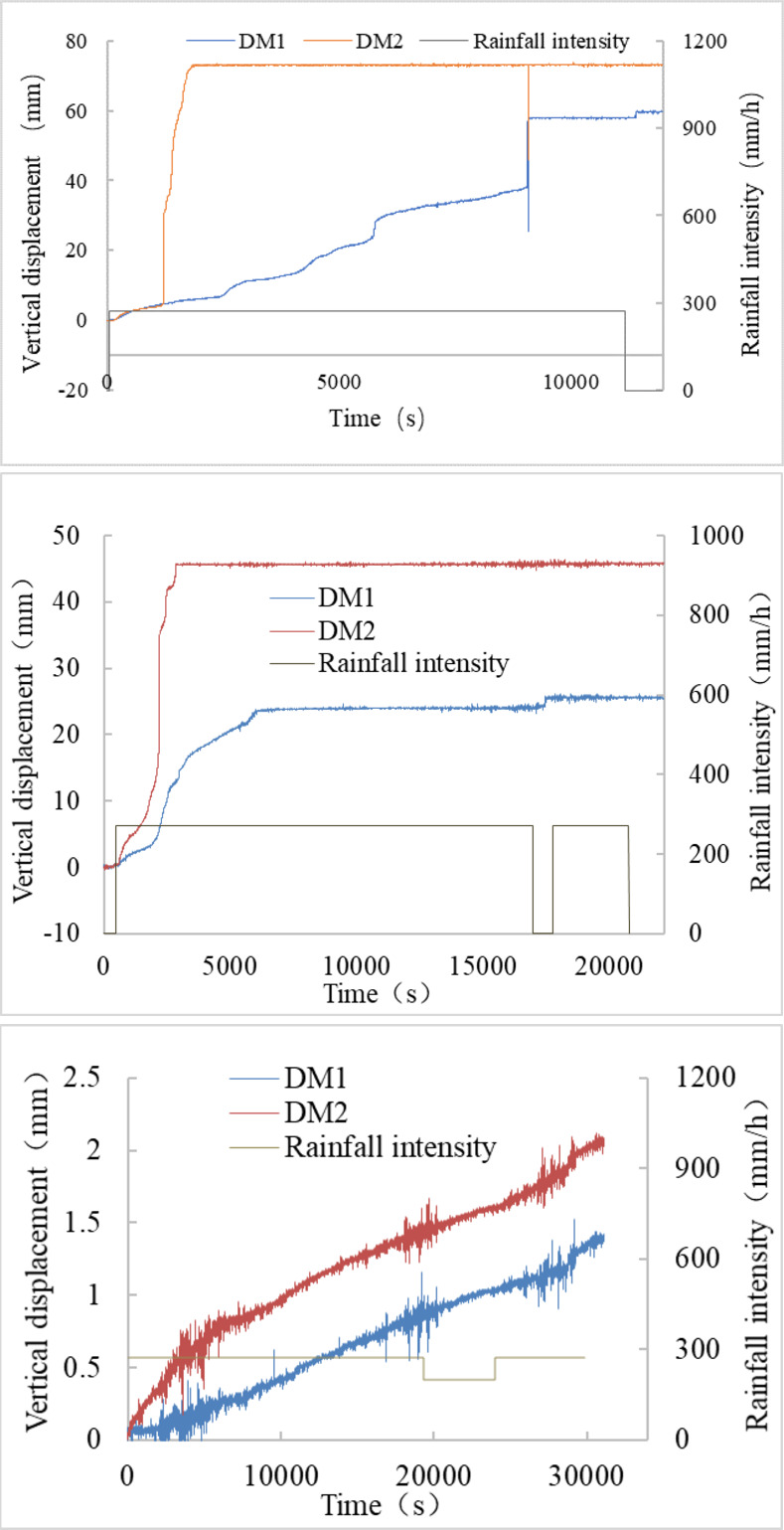
The relationship between displacement, rainfall intensity, and time. (a) S1, (b) S2, (c) S3.

Fig 12 illustrates the relationship between the displacement of measurement sites on the slope surface, rainfall, and time in the experiment on rainfall-induced slope failure. It can be observed that:

- The displacement at the measuring point on the S1 sample is the greatest, followed by the S2 sample, and the S3 soil sample with a 100% coarse-grained content has the smallest displacement. Consequently, as the coarse-grained material increases, the degree of damage to the slope surface decreases.
- The two displacement curves of the S1 sample exhibit two distinct forms. Displacement meter 2 (DM2) is precisely situated on the first connected gully, and the curve features an approaching vertical rise segment. It enters the stage of fast deformation and rapidly sustains damage after a short period of sluggish deformation. The gully mode corresponds to the first type of crack-to-failure mode outlined above. Displacement meter 1 (DM1) is positioned on the slope outside the gully (other places), and the displacement curve exhibited is stepped, consistent with the second type of crack-to-failure method mentioned earlier, known as a progressive backward failure.

- The displacement curves of S2 also take two forms. DM2 is located inside the first gully’s range, and its curvature exponentially climbs. DM1 is placed in other regions, and its deformation curve shows a three-stage rise: a gradual ascent, a logarithmic rise, and then a new equilibrium.
- The slope of S3 has the smallest displacement, with its highest vertical displacement under rainfall being 3.69 mm. The displacement curve has the following shape: gently rising.

### The influence of rainfall and excavation at the toe of the slope on displacement and pore pressure variations

Changes in slope displacement can influence pore pressure under conditions of excavation at the slope’s toe with rainfall. Therefore, when analyzing pore pressure, slope displacement should also be taken into account.

#### Changes in displacement and pore pressure during rainfall infiltration

The permeability coefficient of the soil layer is significantly influenced by the microstructure of the soil. Factors such as porosity, particle size, arrangement, and material hydrophilicity impact soil permeability by affecting the size of the seepage channel, tortuosity of the seepage path, and the number of seepage channels [26–28]. As coarse-grained soils are non-hydrophilic, those with a high coarse-grained component exhibit high porosity. Consequently, their permeability coefficient (k) should be larger than that of stone-free soil. For S1, S2, and S3, the respective permeability coefficient relationship is k1 < k2 < k3.

Applying Darcy’s law to compute vertical seepage, we assume a uniform soil layer and a total head loss denoted as *Δh*. The distance from the slope surface to the location of the pore pressure gauge is represented by *H*. The permeability coefficient, denoted as k, is then given by:

$$k_{i}=vH/\Delta h$$ (1)

In the formula, *ν* represents the permeability rate, while *k*_*i*_ denotes the soil permeability coefficient for the *S*_*i*_ sample.

When *H/Δh* remains constant, the permeability rate of the soil is directly proportional to the permeability coefficient. Additionally, the permeability duration at the same distance is inversely proportional to the permeability coefficient, as per Darcy’s law calculation formula. The greater the soil permeability coefficient, the faster the pore pressure response.

**Fig 13:**
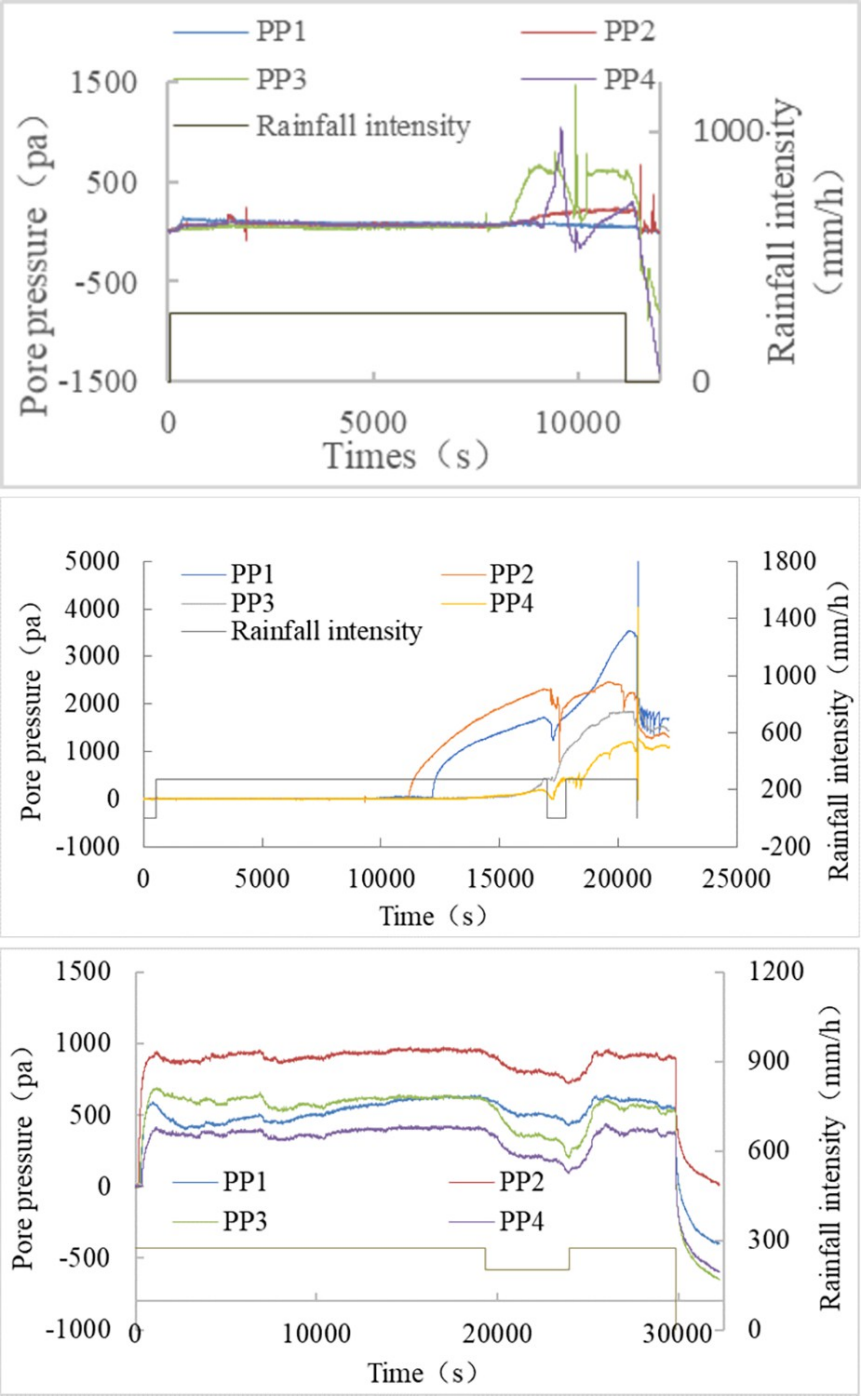
The relationship between pore pressure and rainfall intensity. (a) S1, (b) S2, (c) S3.

The response time is the duration from the start of rainfall to the initiation of a rise in pore pressure sensor readings. When the soil layer thickness remains constant, the expected order of reaction times for each pore pressure sensor is t1 > t2 > t3. However, in actual measurements, the observed order is t2 > t1 > t3. In the experiment, the average time taken for S1 is 8743 seconds, as depicted in Fig 13(A). For S2, the response time of the pore pressure is the longest, averaging 11708 seconds, illustrated in Fig 13(B). Conversely, for S3, the response time of the pore pressure is the shortest. The curve rapidly increases at the 112th second after rainfall, followed by continuous fluctuations with rainfall, as shown in Fig 13(C).

This phenomenon is induced by a shift in seepage distance (H) caused by slope damage and soil erosion during rainfall. The following provides a detailed analysis:

Fig 11 illustrates that the rapid movement stage of DM2 occurs at the 1250^th^ second, with the greatest displacement happening at the 1906^th^ second, resulting in a wide gully formation deep into the soil mass, also visible in Fig 7. In Fig 13, the readings of PP3, placed below the gully-affected area, exhibited the earliest fluctuation and increase at the 8303^rd^ second. At the 2476^th^ second, the first failure step curve in DM1 began to manifest, reaching its maximum value at the 9144^th^ second. This indicates the gradual failure of the slope toe between the 2476^th^ and 9144^th^ seconds. Around the 8801^st^ and 9183^rd^ seconds, pore pressure sensors PP2 and PP4, positioned next to the slope toe, started to rise. The vertical seepage distance (*H*) of the rainfall has been shortened due to the development and growth of gullies, as well as the significant loss of soil at the slope toe. The variations in displacement and pore pressure curves in Fig 5 effectively represent the failure process.

The above test results indicate that: ① The failure of the loess spoil slope begins with the wetting of the surface soil, and once some parts of the loess spoil slope become wet and saturated, the soil in that area may be damaged, and water has not yet penetrated the deep soil layer. ② The time it takes for the rainfall seepage line to reach the preset depth decreases as the soil is lost from the slope surface.

For T2-1, Fig 12 indicates that the DM2 reading entered a rapid growth phase at the 590^th^ second, reaching its maximum value at the 2888^th^ second. The DM1 reading exhibited the first stage of deformation at the 536^th^ second, followed by the second stage at the 1767^th^ second, reaching its maximum value at the 5991^st^ second. Referring to Fig 13, PP2 and PP1 demonstrate a rapid increase at the 11216^th^ and 12249^th^ seconds, respectively, while PP3 and PP4 begin to rise slowly after the 15932^nd^ second.

By integrating the progress chart of the T2-1 experiment in Fig 8, the displacement curve in Fig 12, and the pore pressure curve in Fig 13, it is evident that, although the S2 slope transforms earlier, the depth of failure is shallower than that of the S1 slope. Consequently, while slope failure does influence the time it takes for the rainfall seepage line to reach the preset depth, the degree of impact is lower than in the S1 sample.

The above test results indicate the following: ① The failure mechanism of the loess spoil slope, with a coarse-grained content of 25%, primarily involves shallow failure. ② The loss of soil from the slope surface leads to a reduction in the time it takes for the rainfall seepage line to reach the preset depth. ③ Under rainfall conditions, S2 exhibits a shallower degree of damage than S1. This is also reflected in the earlier rise time of the pore pressure sensor in S1 compared to that in S2.

#### Changes in displacement and pore pressure during slope toe excavation

**Fig 14:**
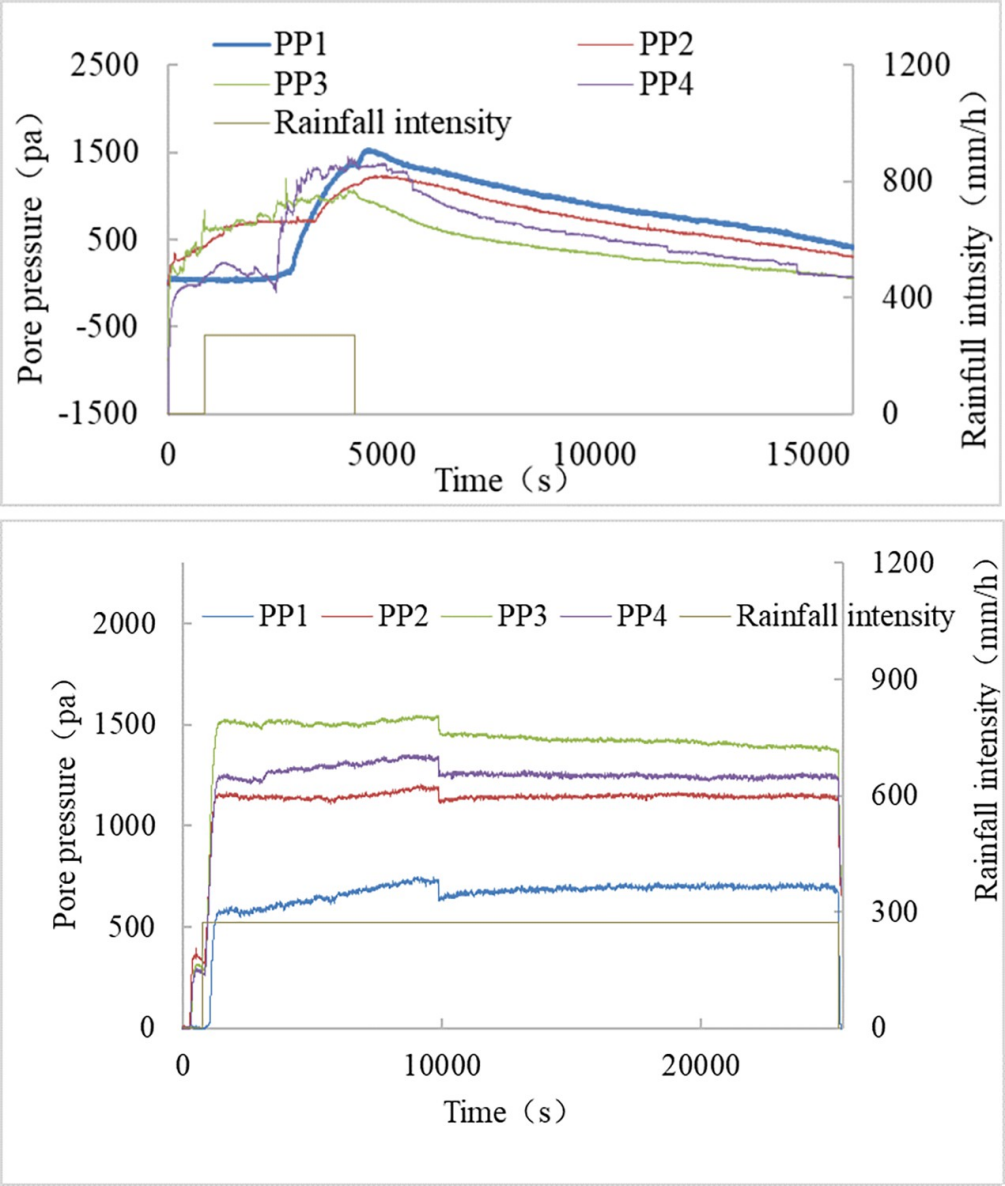
The relationship between pore pressure and rainfall intensity after excavation of slope toe. (a) Test T1-2, S1, (b) Test T3-2, S3.

Excavation of the slope toe has a rapid and significant impact on wet soil. Fig 14 illustrates the relationship between pore pressure and rainfall intensity following the excavation of the slope toe.

T1-2 represents the second stage of the T1 test, following T1-1, when a specific thickness of the soil layer has become saturated. Although the pore pressure sensor readings had changed by the end of the T1-1 test, they had not yet reached their maximum values. This suggests that rainwater had seeped into the area, but the soil had not yet reached saturation.

The displacement meter reading began to increase at 2534 seconds, reaching its peak at 3001 seconds. Concurrently, the pore pressure sensor reading exhibited a notable rise at 2575 seconds following the excavation of the slope’s toe. The response time of the pore pressure sensor has significantly decreased since the initial phase of the experiment, indicating that wet soil requires less time than dry soil for rainfall seepage over the same distance. Immediately post-excavation, the slope experienced failure, suggesting that wet loess spoil is susceptible to rapid damage under continuous rainfall and excavation.

## Conclusions

This study used indoor model tests with loess spoil soil from Xian as the research object to examine the failure evolution process and mode of loose spoil slopes under rainfall and excavation of the slope toe. The complete process and mode are studied based on the results of displacement and pore pressure curves. The following are the primary conclusions:

- There are two distinct types of crack-to-failure evolution modes with three stages of features for the pure loess spoil slope in diverse slope regions under the induction of rainwater: a backward-progressive failing phase in certain sections and a gullying mode in others. Numerous relatively steep slopes (>45°) form, exhibiting loess-specific characteristics, after the landslide stops and stabilizes.
- A three-stage characteristic is evident in the failure process of loess spoil containing 25% coarse-grained material triggered by rainfall. This slope distinguishes itself from pure loess spoil soil slope in that sizable particles remain anchored to the surface, while diminutive loess particles are gradually eroded by rainwater, ultimately resulting in a regional landslide. The extent of maximum damage for this configuration exceeds that of pure loess spoil, although its maximum damage depth is not as substantial. Following the cessation and stabilization of the landslide, the slope angle becomes relatively modest, measuring less than 45 degrees.

- A progressive multi-stage retreat is the slope failure mode when excavating the toe of the slope. The following is the failure process: Transverse tensile cracks first occur, followed by cracks that deepen towards the soil mass, close, and peel off the upper layer of soil around them before accumulating at the slope’s toe.

## Supporting information

S1 Data (ZIP)
